# 408例70岁以上老年肺癌患者的预后因素分析

**DOI:** 10.3779/j.issn.1009-3419.2011.06.05

**Published:** 2011-06-20

**Authors:** 华 郑, 丽 仝, 瑛 胡, 卫华 吴, 红梅 张, 宝兰 李

**Affiliations:** 1 101149 北京，首都医科大学附属北京胸科医院综合科 General Department, Beijing Chest Hospital of Capital Medical University, Beijing 101149, China; 2 101149 北京，首都医科大学附属北京胸科医院肿瘤内科 Department of Medical Oncology, Beijing Chest Hospital of Capital Medical University, Beijing 101149, China

**Keywords:** 肺肿瘤, 老年病学, 预后, Lung neoplasms, Geriatrics, Prognosis

## Abstract

**背景与目的:**

随着人口老龄化，老年肺癌的发病率呈上升趋势。统计数据显示在过去的10年中，70岁以上人群中肺癌发生率及死亡率较前增加。本文以70岁作为老年肺癌的分界线，旨在分析影响其预后的因素。

**方法:**

回顾性分析408例70岁以上老年肺癌患者资料，利用SPSS 13.0统计软件进行单因素及*COX*回归多因素分析，探讨性别、年龄、合并症、症状、病理类型、临床分期、浆膜腔积液、手术、化疗、放疗等因素对生存的影响。

**结果:**

单因素分析显示症状、临床分期、浆膜腔积液、手术、化疗及化疗周期等因素对预后有影响，*COX*回归多因素分析显示，临床分期（*P* < 0.001）、手术（*P*=0.013）、化疗次数（*P*=0.001）为影响预后的因素。

**结论:**

老年肺癌在早期可从手术及术后辅助化疗中获益，在晚期进行至少4周期的化疗可延长生存时间。对于老年肺癌单药化疗方案不失为一个较好的选择。浆膜腔积液特别是心包积液会明显影响预后，应积极控制处理积液。

## Abstract (en)

**Background and objective:**

With the aging of the population, the incidence of lung cancer in elderly rises. It showed that the incidence and mortality of lung cancer in people over 70 years were increased in the past 10 years. We defined age 70 as boundary line of the elderly patients in lung cancer. The aim of this study is to analyze the factors of affecting prognosis.

**Methods:**

A retrospective study had enrolled 408 cases of lung cancer aged over 70 and SPSS 13.0 software was used in univariate analysis and *COX* regression analysis to analyze factors affecting prognosis, such as gender, age, complications, symptoms, pathological type, clinical stage, effusion, surgery, radiotherapy, chemotherapy and so on.

**Results:**

In univariate analysis, symptoms, stage, effusion, surgery, chemotherapy and chemotherapy cycles showed affecting prognosis significantly. In *COX* regression analysis, it showed that clinical stage (*P* < 0.001), surgery (*P*=0.013), chemotherapy cycles (*P*=0.001) were independent prognostic factors.

**Conclusion:**

Elderly lung cancer patients could be benefit from surgery and adjuvant chemotherapy while early stage. At late stage, their survival time may be prolonged when receive chemotherapy at least 4 cycles. Single- agent chemotherapy would be a good choice for elderly lung cancer. Effusion, particularly, pericardial effusion significantly influenced the prognosis, so that it should be effectively controlled.

随着人口的老龄化，老年肺癌的发病率呈上升趋势。统计数据显示在过去的10年中，肺癌的发病率及死亡率在50岁及更年轻的人群中有所降低，而在70岁以上的人群中则增加^[1]^。一项老年肺癌的回顾性分析^[2]^指出，大于70岁的患者化疗相关毒性事件的风险明显高于70岁以下的患者。因此我们以70岁作为老年肺癌的分界线，分析影响70岁以上老年肺癌预后的因素，为临床工作提供理论依据。

## 1 材料与方法

### 1.1 临床资料

选取1998年1月-2002年12月在我院住院治疗、临床资料完整、确诊时年龄在70岁以上的肺癌患者408例进行回顾性分析。其中男性330例，女性78例，年龄范围70岁-93岁，平均年龄（73.59±3.53）岁。无合并症的有159例，合并至少一种疾病的有249例，合并症中较多的是心脑血管疾病、肺结核、消化系统疾病、慢性阻塞性肺疾病及糖尿病等。无症状体检发现肺癌患者32例，以呼吸系统症状（咳嗽、胸闷、胸痛、咯血等）就诊337例，以非呼吸系统症状（发热、体重减轻、纳差、颈部颜面肿胀、声嘶等）就诊39例。鳞癌167例，腺癌140例，腺鳞癌35例，小细胞癌52例，其他（包括大细胞癌、肺泡细胞癌、乳头状腺癌及未分病理类型的肺癌）14例。按照美国癌症联合会（American Joint Commitee on Cancer, AJCC）1997年制定的第5版肺癌TNM分期系统进行临床分期，Ⅰa期-Ⅲa期的患者有173例，Ⅲb期-Ⅳ期的患者有235例。

### 1.2 治疗情况

408例患者中接受治疗244例，未接受任何治疗164例。Ⅰa期-Ⅲa期的患者中接受治疗与未接受任何治疗患者的比例为2.6:1（125/48），Ⅲb期-Ⅳ期的患者中该比例为1:1（119/116）。接受手术治疗的患者93例，其中全肺切除术19例，肺叶切除术、肿块楔形切除术2例，开胸探查5例。接受化疗146例，未做化疗262例；化疗周期 < 4者111例，化疗周期≥4者35例。非小细胞肺癌（non-small cell lung cancer, NSCLC）的化疗方案主要为长春瑞滨+顺铂或卡铂、紫杉醇+顺铂或卡铂、吉西他滨+顺铂或卡铂、紫杉醇单药及长春瑞滨单药等。小细胞肺癌的化疗方案主要为足叶乙甙+顺铂或卡铂、拓扑替康+顺铂或卡铂或足叶乙甙单药、拓扑替康单药化疗等。

### 1.3 生存期

观察终点为死亡或截止日期2007年12月15日。生存期计算是指确诊日期至观察终点日期之间的时间，以月为单位。

### 1.4 统计方法

所有数据用SPSS 13.0软件进行统计分析，生命表法计算生存率及中位生存期，*Kaplan-Meier*法进行单因素分析，*Long-rank*法评价生存差异，*COX*多因素回归模型对所有变量进行分析确定影响预后的因素，*P* < 0.05为差异有统计学意义。

## 2 结果

### 2.1 随访结果

截至2007年12月15日，累计死亡400例。1年、2年、3年、5年生存率分别为41.2%、14.2%、8.3%、1.5%，中位生存时间为10.3个月。

### 2.2 预后因素分析

#### 2.2.1 单因素分析

对性别、年龄、合并症、症状、病理类型、临床分期、浆膜腔积液（包括胸腔积液和心包积液）、手术、化疗、放疗等情况进行单因素分析（表 1），结果显示症状、临床分期、浆膜腔积液、手术、化疗及化疗周期是影响预后的因素（*P* < 0.05），性别、年龄、病理类型及放疗不影响预后。联合化疗不优于单药化疗（*P*=0.545）。合并症的多少不影响生存（*P*=0.405）（图 1）。Ⅲb期患者治疗与否生存期分别为15.93个月与7.51个月（*P* < 0.001），Ⅳ期为10.11个月与5.08个月（*P* < 0.001）（图 2）。Ⅲb期患者中是否有浆膜腔积液生存期存在差异（*P*=0.01），而Ⅳ期患者中有无浆膜腔积液对生存无影响（*P*=0.819），有心包积液患者的生存期明显少于无心包积液的患者（*P*=0.011）和无积液者（*P* < 0.001）（图 3）。

**1:** 老年肺癌患者单因素分析 Univariate analysis of lung cancer patients in the elderly

Characteristics		*n*(%)	MST (month)	*P*
Sex	Male	330 (80.9)	14.02	0.772
	Female	78 (19.1)	13.12	
Age (year)	70-75	311 (76.2)	14.20	0.284
	≥76	97 (23.8)	12.61	
Symptoms	No	32 (7.8)	18.50	0.047
	Yes	376 (92.2)	13.42	
Histological type	NSCLC	356 (87.3)	14.37	0.061
	SCLC	52 (12.7)	10.08	
Stage	Ⅰ	70 (17.2)	26.35	< 0.001
	Ⅱ	30 (7.4)	17.58	
	Ⅲa	73 (17.9)	14.18	
	Ⅲb	111 (27.2)	11.61	
	Ⅳ	124 (30.4)	7.72	
Effusion fluid	Yes	107 (26.2)	8.47	< 0.001
	No	301 (73.8)	15.71	
Surgical resection	Yes	93 (22.8)	25.81	< 0.001
	No	315 (77.2)	10.41	
Chemotherapy	Yes	146 (35.8)	16.02	0.007
	No	262 (64.2)	12.61	
Radiotherapy	Yes	100 (24.5)	14.31	0.380
	No	308 (75.5)	13.64	
Chemotherapy cycles	< 4	373 (91.4)	13.15	0.003
	≥4	35 (8.6)	21.08	
Chemotherapy regimen	Combination	128 (31.4)	15.63	0.545
	Single agent	16 (3.9)	17.82	
MST: middle survival time.				

**1:**
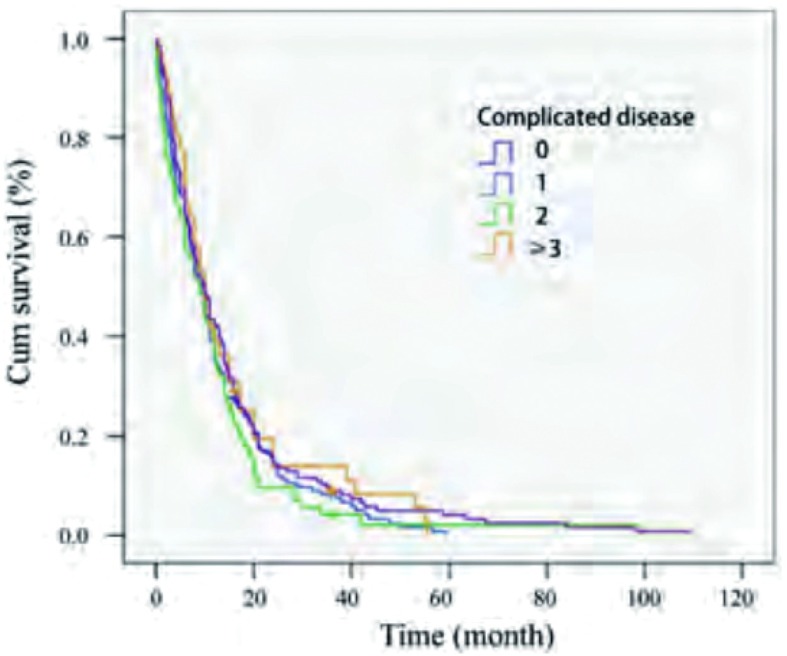
老年肺癌不同合并症数目生存曲线 The survival curve of complications in el-derly lung cancer patients (*P*=0.405)

**2:**
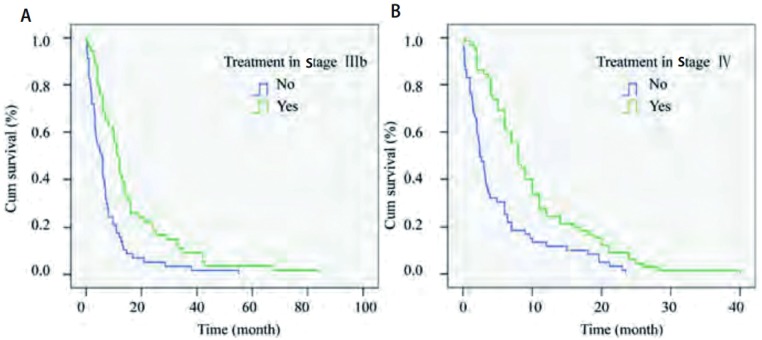
晚期老年肺癌治疗与否对生存的影响。A：治疗与否对Ⅲb期患者生存的影响（*P* < 0.001）；B：治疗与否对Ⅳ期患者生存的影响（*P* < 0.001）。 Influence of treatment to survival in advanced elderly lung cancer patients. A: Influence of treatment to survival in stage Ⅲb (*P* < 0.001); B: Influence of treatment to survival in stage Ⅳ (*P* < 0.001).

**3:**
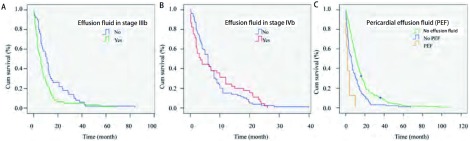
晚期老年肺癌中积液对生存的影响。A：Ⅲb期积液对生存的影响（*P*=0.01）；B：Ⅳ期积液对生存的影响（*P*=0.819）；C：心包积液对老年肺癌生存的影响（*P* < 0.05）。 Influence of effusion fluid to survival in advanced elderly lung cancer. A: Influence of effusion fluid to survival in stage Ⅲb (*P*=0.01); B: Influence of effusion fluid to survival in stage Ⅳ (*P*=0.819); C: Influence of pericardial effusion fluid to survival in elderly lung cancer (*P* < 0.05).

#### 2.2.2 *COX*回归模型多因素分析

将单因素分析中有统计学意义的变量放入*COX*回归模型进行多因素分析，结果显示临床分期、手术、化疗次数为影响预后的因素（表 2）。

**2:** 老年肺癌*COX*回归多因素分析 *COX* regression of variables in the equation of lung cancer in elderly

Variable	B	SE	Wald	df	*P*	Exp(B)	95%CI for Exp (B)
Symptom	0.060	0.143	0.175	1	0.676	1.061	0.803-1.404
Stage	0.256	0.044	34.407	1	< 0.001	1.292	1.186-1.407
Effusion fluid	0.009	0.129	0.005	1	0.945	1.009	0.784-1.299
Surgery	-0.208	0.084	6.199	1	0.013	0.812	0.689-0.957
Chemotherapy	0.134	0.122	1.212	1	0.271	1.144	0.900-1.453
Chemotherapy cycles	-0.226	0.070	10.417	1	0.001	0.798	0.696-0.915

## 3 讨论

我国肺癌的发病率呈逐渐上升趋势，2015年我国将成为全球肺癌发病数最多的国家^[3]^，老年肺癌的发病率也会随之增加。虽然有研究^[4]^显示年龄不是肺癌的独立预后因素，但由于老年患者各脏器功能减退，合并症及治疗相关并发症的发生增多，常使老年人不能顺利完成治疗或放弃治疗，从而影响老年肺癌患者的生存期。

本次研究中408例老年肺癌仅224例患者接受了至少一种治疗措施，而164例患者未接受任何治疗，晚期放弃治疗的患者所占比例较早期明显增加。本次研究显示治疗可以延长老年肺癌生存期，特别是在晚期差异更为明显，而且各种合并症并不影响生存。多项研究^[5-7]^表明老年肺癌患者的生存期与非老年肺癌患者相比并无差异。所以对于老年肺癌患者，无论处于早晚期，当体力状况允许时应进行治疗，可以改善生存情况。

单因素分析中不同就诊原因的患者生存期存在差异，其中无症状体检发现者生存期长。究其原因，大多无症状体检发现者属于疾病早期，生存期较长，而出现临床症状就诊者多有病变阻塞气道、胸腔积液压迫肺叶或肿大淋巴结压迫上腔静脉等症状，这种情况多数已属肺癌晚期，故生存期较短。可见不同就诊原因的患者生存期存在差异是分期早晚所致，因此，在多因素分析中不同就诊原因的患者生存期没有差异（*P*=0.676），而单因素分析和多因素分析中肺癌分期均为影响预后的因素（*P* < 0.001），该结果与文献^[8-10]^报道一致。

有无癌性浆膜腔积液对生存期也有影响，这与分期有一定关系，因为只有晚期患者才有各种癌性积液。本次研究显示Ⅲb期患者中有无积液生存期存在差异（*P*=0.01），而Ⅳ期患者中有无积液对生存无明显影响（*P*=0.819）。Ⅳ期患者存在远处转移，包括头、肝、骨、肾上腺等器官，可导致昏迷、肝衰竭、骨折、内分泌电解质紊乱，从而缩短生存期，其影响程度与是否存在浆膜腔积液相似；而Ⅲb期患者仅存在局部病变，浆膜腔积液的存在会明显影响患者的生活质量，并导致呼吸循环衰竭而缩短生存期，因此在2009年新TNM分期^[11]^中将积液划分至M1a期，与我们本次的研究结果符合。

有心包积液者的生存期明显少于无心包积液（*P*=0.011）和无积液者（*P* < 0.001），说明心包积液对预后的影响比胸腔积液明显。文献^[12, 13]^报道心包积液为预后差的标志，而且心包积液中查到肿瘤细胞预后更差，与我们的结果一致。临床上少量的心包积液即会明显影响患者生活质量，大量心包积液会导致心包填塞危及生命，胸腔积液的危害不及心包积液。因此，当患者出现浆膜腔积液时需积极引流，缓解症状，必要时浆膜腔内注药减少渗出，从而延长生存时间。

小细胞肺癌与NSCLC的生存期没有差异（*P*=0.061）。没接受任何治疗者，不同病理类型的生存期没有差异（*P*=0.955），而接受治疗者不同病理类型患者的生存期存在明显差异（*P* < 0.001），说明小细胞肺癌的预后较NSCLC差，虽然治疗后生存获益，却不如NSCLC获益程度大。小细胞肺癌较凶险，进展迅速，但对化疗、放疗均敏感，因此治疗后可迅速缩小，但复发较快，难以长期控制疾病，而NSCLC虽对化疗、放疗的疗效不及小细胞肺癌迅速和明显，但疾病控制时间相对较长，从而延长患者的生存期，无论哪种病理类型，均可从治疗中获益。

不同的治疗方式对预后也有明显的影响。单因素分析中手术及化疗对预后有影响（*P* < 0.05），而放疗对预后影响不明显（*P*=0.38），可能与放疗仅解决局部，而不能控制全身病情进展有关。在综合治疗中手术加化疗的患者生存期最长（30.36个月），比单纯手术患者（26.91个月）延长3.45个月，因此提倡早期老年肺癌患者术后进行辅助化疗。在多因素分析中化疗与否并未显示为独立预后因素，考虑是因为化疗周期的多少比是否化疗对预后影响更明显。化疗周期≥4周期的患者生存期明显长于化疗 < 4周期的患者（*P*=0.003）。因此，无论是早期术后辅助化疗，还是晚期患者进行一线化疗，均建议化疗4周期以上。这点也与2010年NCCN指南相符。

目前，老年肺癌的化疗方案也是颇具争议。为减轻老年肺癌化疗毒副反应，目前第三代细胞毒药物的单药化疗方案已成为热门研究方向。一项Ⅱ期临床研究^[14]^显示，在≥70岁的老年晚期肺癌中长春瑞滨联合顺铂方案的生存期优于长春瑞滨单药化疗方案（*P*=0.030），但相应的毒副反应也明显增加（*P* < 0.01）。而另一项研究^[15]^显示吉西他滨单药化疗与含铂联合化疗方案相比生存期无差异（*P* > 0.05），且单药方案耐受较好。第三代细胞毒药物单药化疗方面的研究还很多^[16-19]^，包括长春瑞滨、吉西他滨、紫杉醇类及培美曲塞等，而它们的疗效相比并没有明显差异^[20, 21]^，且耐受性较好，甚至PS评分差的患者也可耐受单药化疗^[16]^。2010年ASCO会议上有一项研究^[22]^引起高度重视，该研究包括451例70岁-89岁（中位年龄77.2岁）、体能状态（PS）评分为0-2的Ⅲ/Ⅳ期NSCLC患者，结果显示与吉西他滨或长春瑞滨单药治疗相比，卡铂（月疗）+紫杉醇（周疗）联合化疗可明显改善患者总生存时间（10.4个月*vs* 6.2个月，*P*=0.000, 1）和无进展生存期（6.3个月*vs* 3.2个月，*P* < 0.000, 1），但联合组的3/4级血液学毒性反应更常见，中性粒细胞减少症发生率分别为54.3%和14.3%。本次研究含铂联合方案与单药方案相比生存期没有差异（*P*=0.545），甚至单药方案的生存期比联合方案生存期略有延长。因此，可根据患者体能状态、生存期预计时间等因素来选择单药或联合方案化疗。

总之，老年肺癌患者在早期可从手术及术后辅助化疗中获益，在晚期进行至少4周期的化疗可延长生存时间。老年肺癌患者采用单药化疗方案不失为一个很好的选择。浆膜腔积液，特别是心包积液会明显影响预后，应积极控制处理积液。
